# P389: A rapid biological indicator for sterility assurance

**DOI:** 10.1186/2047-2994-2-S1-P389

**Published:** 2013-06-20

**Authors:** PE Gordon

**Affiliations:** 1 Germgard Lighting LLC, Greenwich, CT, USA

## Introduction

The use of endospore based industry standard Biological Indicators has been accepted practice for establishing that a sterilization process has been effective in sterilizing a given batch of surgical instruments in acute care facilities and medical device manufacturing for over 30 years. Although they have been applied to verifying the efficacy of instrument sterilizers such as autoclaves (steam), H_2_O_2_, O_3_ + H_2_O, etc, they have not yet been applied to verification of other disinfection processes such as Ultraviolet light (UVC). No such standard Biological Indicator system is readily available for disinfection assurance of drinking or waste water, of food containers on conveyor lines, or on important surfaces in hospital rooms after exposure to disinfecting UVC.

Such a Biological Indication capability provides a major improvement in sterilization and disinfection practice and utility, and potentially results in more certain sterilization and disinfection assurance results than current practice. Products based upon such a technique would add a reliable testing, compliance adherence, and risk management component to sterilization and disinfection tasks, greatly improving on current practice.

## Objectives

A bio-assay technique based DPA-triggered Tb^3+^ luminescence is described. It potentially can be an excellent approach for counting endospores that have survived steam or gas sterilization, and UVC disinfection, and verifying efficacy. The technique has the potential to accurately confirm sterilization or disinfection within 15 minutes.

## Conclusion

Such a Biological Indication capability provides a major improvement in sterilization and disinfection practice and utility.

## Disclosure of interest

None declared.

